# A conceptual framework for assessing behavioral flexibility of species in response to extreme climatic events

**DOI:** 10.1038/s41598-023-45756-2

**Published:** 2023-10-28

**Authors:** Eric I. Ameca, Lucy Chamart, Paul A. Garber

**Affiliations:** 1 Key Laboratory for Biodiversity Science and Ecological Engineering, Beijing Normal University, Beijing, China; 2 Climate Change Specialist Group, Species Survival Commission, International Union for Conservation of Nature, Gland, Switzerland; 3 International Centre of Biodiversity and Primate Conservation, Dali University, Dali, Yunnan China; 4 Department of Anthropology and Program in Ecology, Evolution, and Conservation Biology, University of Illinois, Urbana, IL USA

**Keywords:** Behavioural ecology, Biodiversity, Climate-change ecology, Conservation biology

## Abstract

Inherent differences in the adaptive capacity of species to flexibly respond to extreme climatic events (ECEs) represent a key factor in their survivorship. We introduce and apply a conceptual framework linking knowledge about species’ current ecology and biology with variation in behavioral flexibility to ECEs. We applied it to 199 non-human primate species currently exposed to cyclones across the global tropics. Our findings suggest that species characterized by an increased ability to exploit a broad range of food types, social systems that permit subgrouping, and habitat types that span a range of environmental conditions may have greater success in coping with cyclones than more narrowly constrained or less adaptable primates. Overall, 15% of species, predominantly of the families Atelidae and Cercopithecidae, were assessed as having high or very high flexibility. In contrast, ~ 60% of primates were assessed with low or very low flexibility. These were species mainly belonging to the Cheirogaleidae, Lemuridae, Lepilemuridae, and Indriidae. While much work remains to better understand mechanisms driving differences in behavioral flexibility of species exposed to extreme climate across vertebrate lineages, our framework provides a workable approach that can improve estimates of current vulnerability to these phenomena and better inform conservation and management strategies.

## Introduction

Environmental changes caused by natural climate variability, including extreme climatic events (ECEs), play a key role in shaping the earth’s biodiversity at multiple levels^1,2^. Recent evidence suggests that ECEs may result in severe negative effects on animal populations including extensive habitat disruption, critical reductions in food availability, and reduced survivorship^3^. Given that global temperature increases are causing more frequent and more intense ECEs^4^, quantifying patterns of species’ exposure and identifying behavioral strategies that enable species to avert the worst impacts of these events are critical conservation imperatives^5–7^.

The vulnerability of species to climate change is related to several factors including the type of climatic event, its frequency, intensity, duration, and the ability of the local environment to recover^8,9^. Vulnerability also is mediated by intrinsic biological attributes that shape species’ capacity to anticipate, withstand, and adjust physiologically and behaviorally to these disruptive environmental changes^10^. Attributes associated with phenotypic plasticity in diet, foraging strategies, patterns of habitat utilization, social organization, and problem-solving may reduce the potentially adverse consequences on individual survival and population persistence during or in the aftermath of ECEs. For instance, phase shifting between diurnal and nocturnal activity cycles in white-tailed deer (*Odocoileus virginianus*) has been linked to increased access to temporal refugia, reducing exposure to thermal extremes^11^. Similarly, species characterized by increased mobility and dietary adaptability are expected be less affected by ECEs by opportunistically exploiting altered conditions in their current habitat or by ranging into new or neighboring habitats. In this regard, Hispaniolan Parrots (*Amazona ventralis*) were found to move into inland broadleaf forest habitats at higher elevation when coastal scrub forests were heavily damaged by cyclone Georges^12^. Flexibility in habitat use in the face of ECEs has been observed in a range of taxa such as wetland birds, island bats, and sharks^13–15^. Finally, Mexican spider monkeys (*Ateles geoffroyi vellerosus*) have been observed to adjust patterns of social cohesion and grouping in response to the aftermath of ECEs. Individuals were reported to spend less time fused into a large group and increased time traveling in small, scattered subgroups post-cyclone; this shift appeared to reflect a foraging strategy designed to optimize food intake across a landscape characterized by a marked reduction in food availability caused by ECEs^16^.

Significant progress has been made in documenting and characterizing species behavioral responses to changes in average climatic conditions in the field and the laboratory (e.g., changes in temperature, precipitation, and wind speed) over different time frames (weeks, months, years) across several vertebrate lineages^17^. Despite this progress, we lack a general framework to explicitly inform which species exposed to ECEs may be more susceptible to adverse consequences due to insufficient behavioral flexibility to cope with changing environmental conditions. Having this information alongside knowledge of the species’ current risk of becoming extinct (e.g., in terms of IUCN Red List status) can be valuable for implementing and prioritizing effective management and conservation policies, especially among threatened taxa.

**Figure 1:**
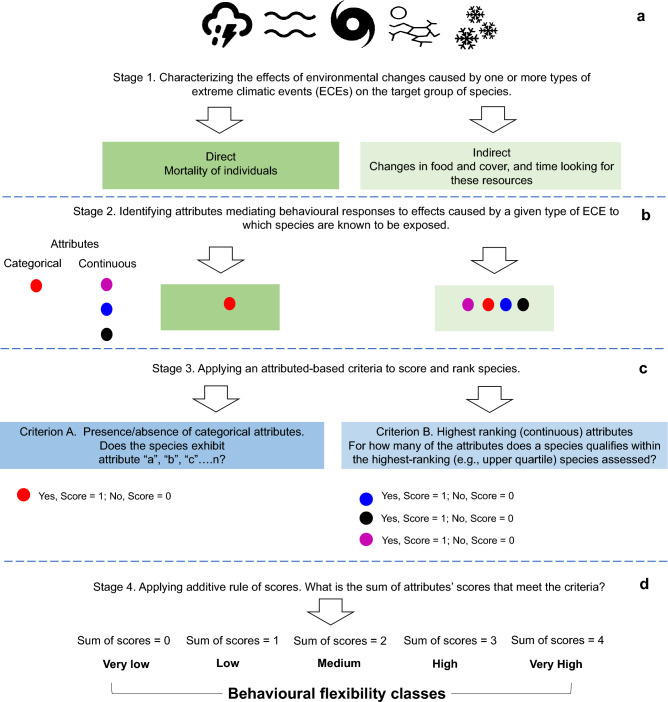
Conceptual framework for assessing species behavioral flexibility in response to extreme climatic events. Behavioral flexibility is assessed as a function of intrinsic biological and behavioral attributes (e.g., habitat breadth, diet breadth, elevational range, and fission-fusion grouping pattern) that shape an individual’s capacity to anticipate, withstand and/or effectively adjust their behavior before, during and/or in the aftermath of extreme climatic events (e.g., cyclone, flood, heatwave).

Here, we present a conceptual framework (Fig. 1) from which to integrate knowledge of species’current exposure to ECEs with characteristics associated with species’ behavioral flexibility. We apply this framework to 199 species of nonhuman primates (hereafter primates) naturally exposed to severe weather events in the form of cyclones (e.g., hurricanes and typhoons). We focus on primates because they represent the most threatened taxonomically diverse group of mammals (primates represent the 3^rd^ most speciose mammalian order), with 69% of 522 species, for which data are available, listed as Vulnerable, Endangered or Critically Endangered in the IUCN Red List of Threatened Species, and 93% with declining populations^18^. Primate species vary considerably in diet, patterns of habitat utilization, social organization, group size, life history, and problem-solving skills. Identifying relationships among primate species’ differences in behavioral flexibility and exposure to ECEs offers critical insights into understanding the effects of climate change on other animal species.

## Methods

### Spatial information for assessing exposure to cyclones for primates

Compared to other mammals, primates, followed by bats and rodents, have had the greatest exposure to cyclones^19^. This is due to the fact primate distributions (principally the tropics and subtropics) and cyclone formation and landfall (between latitudes 40° S to 40° N except in the southern Atlantic) overlap significantly. A recent study reported that 38% of primate species worldwide are vulnerable to ECEs^20^.

We extracted the distribution maps of 426 primate species and subspecies (for simplicity here we refer to these collectively as primate species) that are available as shapefiles, from the IUCN’s online repository of spatial data for terrestrial mammals^21^. Cyclone spatial information was obtained from the International Best Track Archive for Climate Stewardship (IBTrACS)^22^. With a 0.1° spatial resolution, IBTrACS version 4.0 is the most complete collection of cyclone tracks to date.

### Quantifying exposure to cyclones

We calculated contemporary cyclone exposure in areas within each species distribution identified as “extant” following the IUCN mapping standards^23^. Extant areas represent localities with current or recent records, up to ~ 30 years, and where suitable habitat for species at appropriate altitudes remains. We focused the exposure analysis to the period from January 1990 to December 2021 in areas where primate populations are most likely to occur (IUCN 2019). Using the *intersect* function in ArcMap^24^, version 10.7, we counted the number of times that individual cyclone tracks overlaid any portion of the primates’ extant distribution. Primate species intersected by one or more cyclone trajectories were assessed as exposed. Consequently, we included those species in applying our framework (Fig. 1).

### Behavioral flexibility framework and implementation

The first step in the framework (Fig. 1a) requires knowledge of the pathways through which ECEs can exert negative impacts on the target species. Accordingly, we reviewed the literature on the effects of cyclone ecology on forest ecosystems^25,26^ to understand how these weather events generate direct and indirect negative impacts on animal species (Fig. 1a). For primates, the direct consequences of strong winds and precipitation caused by cyclones can have an immediate impact on mortality as individuals are injured falling out of trees or struck by falling tree branches^27–30^. Indirect consequences of a cyclone event relate to an initial loss of food, downed trees leading to a loss of refuge and sleeping sites, the loss of a continuous tree canopy leading to increased time spent traveling on the ground, as well as adjustments in behavior (time and energy) invested in locating resources post-cyclone^16,30,31^. On the basis of this knowledge, the second stage involves reviewing species’ attributes associated with behavioral responses to climate change impacts^9,17,32^, and identifying traits relevant for the target species under exposure to cyclone disturbance (Fig. 1b). Our search resulted in three continuous attributes “diet breadth”, “habitat breadth”, “elevational range”, and one categorical attribute, “a fission–fusion grouping pattern” (Table 1). We extracted information on these four species attributes from the literature pertaining to primate natural history and ecology from the “Handbook of Mammals of the World (2013)”, and “All The World’s Primates (2016)”. In addition, we supplemented our search using six online databases: Animal Diversity Web, Encyclopedia of Life, IUCN Red List of Threatened Species, Lemurs of Madagascar, Madagascar Lemurs Portal, and New England Primate Conservancy (See Supplementary Appendix S1 online). Diet breadth was defined as the number of different food types consumed by the species. We adapted PanTHERIA’s classification of food types for primates as follows: Vertebrate, Invertebrate, Fruit, Flowers/Nectar/Pollen, Leaves/Branches/Bark/Exudates, Seeds, Fungi, and Soil. Habitat breadth was defined as the number of habitat types the species is known to exploit. Habitat types were available from the IUCN Red List assessment of each species which follows the IUCN habitat classification scheme (Version 3.1). Elevational range was extracted from the upper and lower elevation limits (in meters) reported for the species. Finally, species that form subgroups or cliques that temporarily forage, rest, or travel independently but coalesce to form a larger cohesive group during the majority of the day and at night, are considered to have a fission–fusion grouping pattern. The detailed rationale for the attributes selected is shown in Table 1.

**Table 1:** Species’ attributes associated with behavioral flexibility in response to cyclone-induced disturbance.

Attributes	Rationale
Diet breadth	Having the capacity to exploit multiple food types may help individuals cope with episodes of a severe reduction in food availability caused by cyclone disturbance (e.g., wind, precipitation, flooding, landslides, flower or fruit abortion) in the months following the event. Primate species with greater flexibility in the choice of food types are expected to be less impacted by these events
Habitat breadth	Primate species that can successfully exploit a broad range of habitat types such as primary forest, disturbed forest, evergreen forest, broadleaf forest, woodlands, savannas, are expected to locate a greater number of safe refuges (e.g., rock crevices, tree holes, dense bushes, vine tangles, caves, the trunks of large trees, areas of continuous forest) that enable them to survive against the strong winds, heavy precipitation, and habitat disruption resulting from a cyclone. This is especially crucial if preferred refuges are disrupted or destroyed
Fission–fusion grouping pattern	Primates exhibiting fission–fusion dynamics have the flexibility to breakdown into small and dispersed subgroups for purposes of locating scarce, scattered and randomly located food patches in the aftermath of a cyclone. It may take several months to years before pre-cyclone levels of food productivity are restored
Elevational range	Primate species that have the flexibility to occupy a larger altitudinal gradient are more likely to encounter a greater number of habitat types and food resources post cyclone than species that range within a narrow altitudinal band

Attribute data completeness and the presence of missing values can affect the reliability of individual assessments of behavioral flexibility. For species lacking data values for one or more attributes, and assuming marked overlap in the ecology and behavior of closely related species, completeness can be improved by using information from congeners^9^. We followed this approach by calculating the mean value of all data available for a missing attribute from congeneric species. This procedure may render some assessments less robust compared with cases in which all attribute data are derived from a single species. To account for this difference, we adopted a measure of confidence in each attribute score based on data availability (Table S1). Confidence levels (poor, moderate, good) were then used to calculate the reliability of the behavioral flexibility class (See below) to which each primate species was assigned. Overall, data completeness for the attributes of fission–fusion, habitat breadth, and diet breadth was high (98%, 95%, and 95%, respectively, of the total number of primate species assessed). Elevation range data were the least complete attribute (65% of total species assessed).

In the third stage of the framework (Fig. 1c), attributes were scored to create a species rank, reflecting their values for each attribute relative to all other species under assessment. For categorical attributes, we applied criterion “A” related to the presence of that attribute and a score of “1” was assigned. If the attribute was absent, we assigned a score of “0”. For continuous attributes we applied criterion “B” in which species with the highest rank for that attribute received a score of “1”. Species with the lowest rank, received a score of “0”. Given that the remaining three attributes were described by continuous variables and ranked using a lower–upper ranking approach, we assigned a score of “1” for each attribute when its value reached the upper quartile and a score of “0” if it fell below the upper quartile. It follows that a primate species that satisfied the criteria for one, two, three or all attributes under investigation was assigned a score of “1”, “2”, “3” or “4”, respectively. A species that did not meet criteria “A” and “B” for any of the attributes was assigned a score of “0” for each attribute. Finally, we applied an additive rule of scores (Fig. 1d) and identified species as having “Very Low” (Score = 0), “Low” (Score = 1), “Medium” (Score = 2), “High” (Score = 3) or “Very High” (Score = 4) behavioral flexibility based on the total number of attributes that met the criteria for “A” and “B” (See working examples in Supplementary Appendix S2 online).

## Results

### Distribution of primate species exposed to cyclones

**Figure 2:**
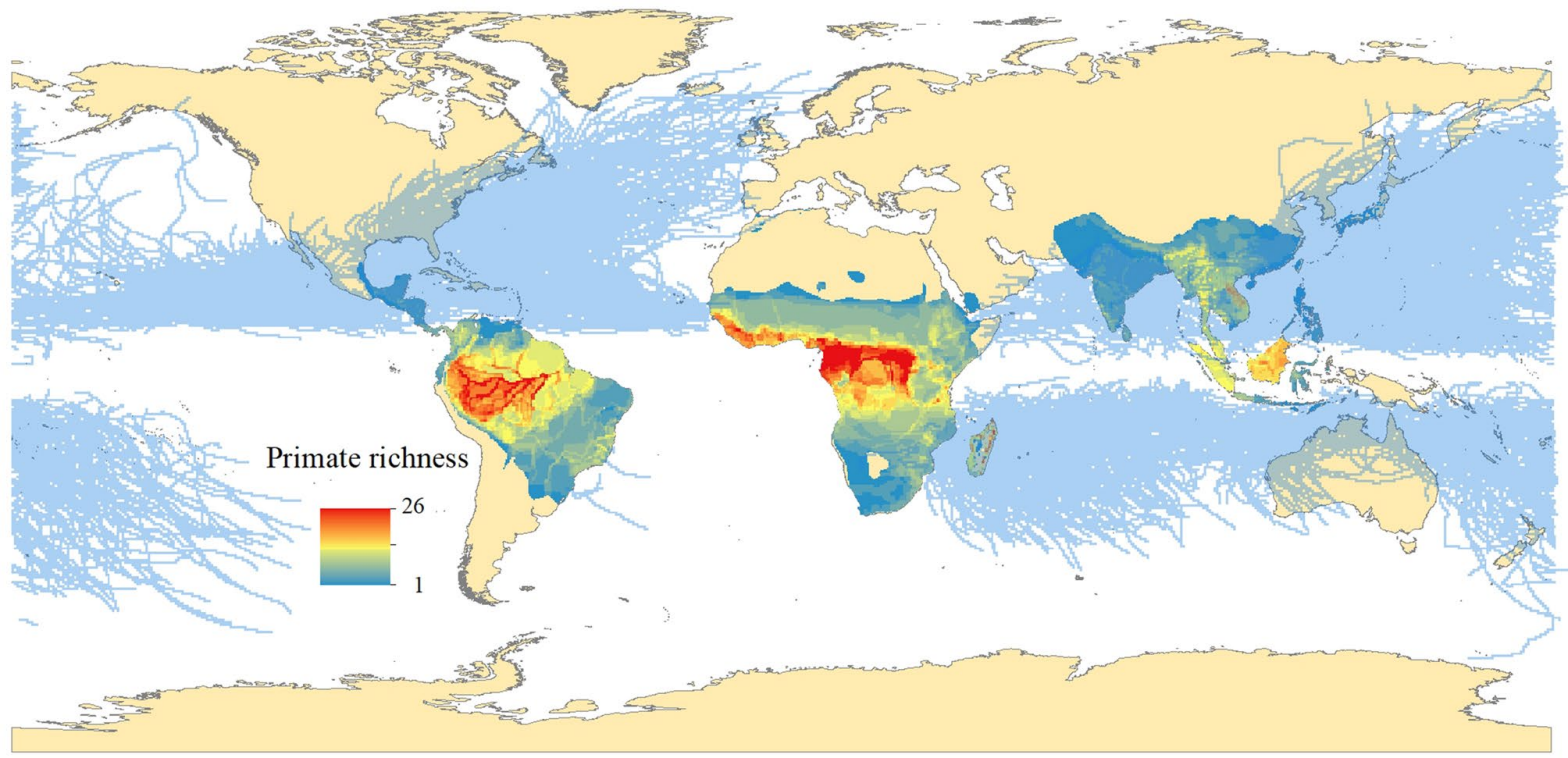
Contemporary exposure of non-human primates to cyclones. Panel shows the distribution of non-human primates (n = 426) and the global distribution of cyclone tracks (represented in blue) from January 1990 to December 2021. Cyclone-exposed primate taxa (n = 199) were selected to implement the framework for assessing behavioral flexibility in response to extreme climatic events.

Based on the present distribution of primate species and the location of individual cyclone events for the period 1990–2021, we identified 199 species as cyclone-exposed (Fig. 2). Most cyclone-exposed primates were found predominantly, but not exclusively, in Madagascar, the Indian subcontinent in Asia, and Central America.

### Behavioral flexibility of primates exposed to cyclones

**Figure 3:**
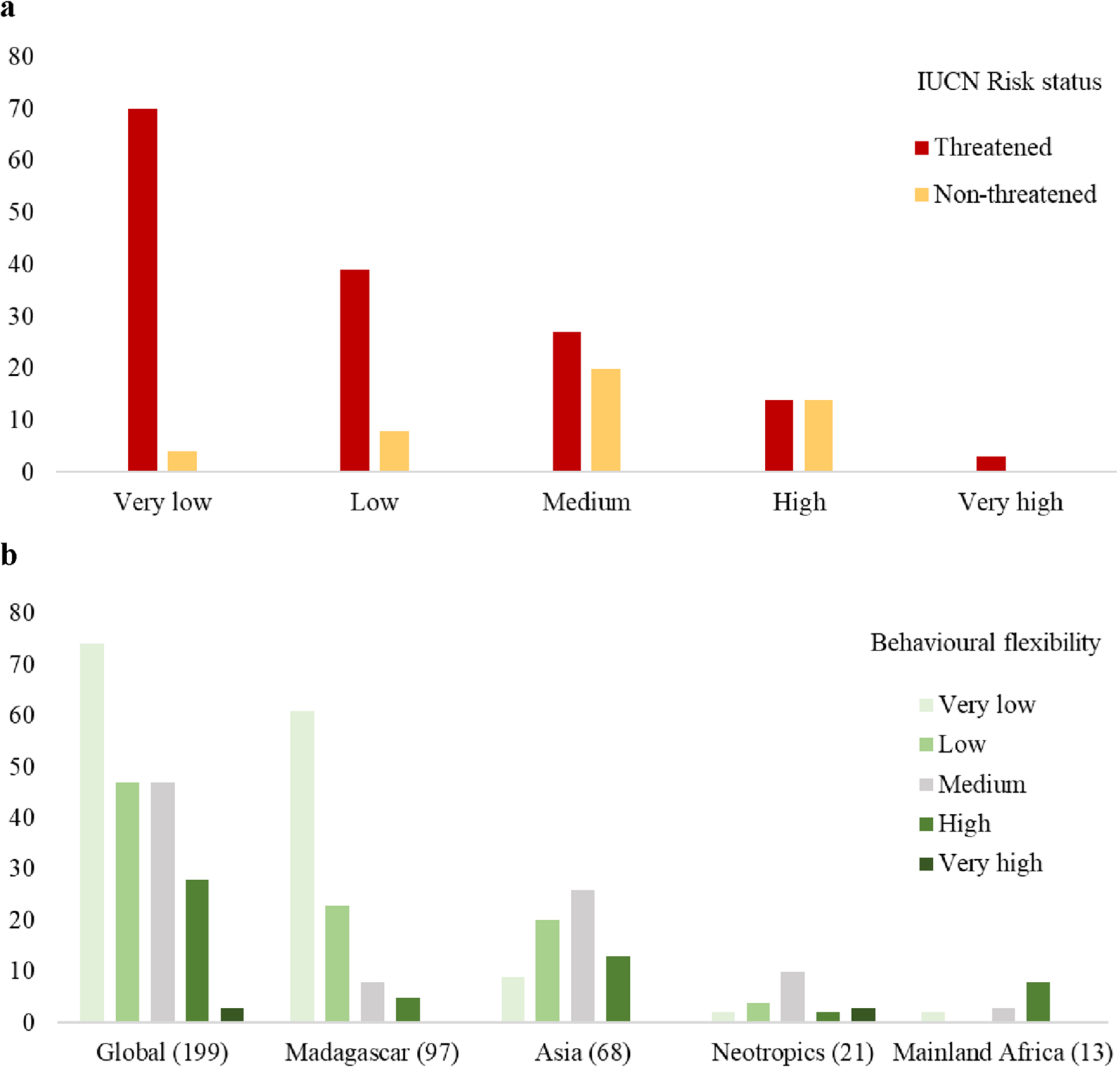
Behavioral flexibility of primate species exposed to cyclones. Panel (**a**) shows the number of cyclone-exposed primate species (n = 199) in each behavioral flexibility category, classified as “threatened” (critically endangered, endangered, vulnerable) or “non-threatened” (near threatened, least concern) by the IUCN in 2022. Panel (**b**) shows primate species in each behavioral flexibility category across the major primate regions. The number of species assessed in each region is given in brackets.

We assessed the behavioral flexibility of 199 cyclone-exposed primate species. Approximately 37% were categorized as having very low and ~ 23% as having low behavioral flexibility (mainly belonging to the Cheirogaleidae [1 species of *Allocebus*, 4 of *Cheirogaleus*, 15 of *Microcebus*, 2 of *Mirza*, and 4 of the genus *Phaner*], Lemuridae [8 species of *Eulemur*, 4 of *Hapalemur*, 1 of *Prolemur*, and 1 of *Varecia*], Lepilemuridae [26 species of *Lepilemur*], Indriidae [9 species of *Avahi,* 8 of *Propithecus*, and 1 of *Indri*] and Cercopithecidae [17 species of the subfamily Colobinae of the genus *Presbytis*, *Pygathrix*, *Rhinopithecus*, *Semnopithecus* and *Trachypithecus*], and 1 species of Cercopithecinae [*Macaca fuscata*]). Species with moderate behavioral flexibility (~ 23%) mainly belonged to Cercopithecidae (6 species of Cercopithecinae [genus *Macaca*], 12 of Colobinae [1 species of *Colobus*, 1 species of *Nasalis,*2 species of *Presbytis*, 1 species of *Pygathrix*, 4 species of *Semnopithecus* and 3 species of *Trachypithecus*]), Hylobatidae (2 species of *Hylobates* and 1 of *Nomascus*), and Lemuridae (2 species of *Eulemur,* 3 of *Hapalemur*, and 1 of *Varecia*). Finally, we found that ~ 14% (n = 28) of these primate species exhibited high and ~ 2% (n = 3) very high behavioral flexibility. This species were mainly cercopithecines (1 species of *Cercopithecus,* 2 species of *Chlorocebus,* 5 species of *Macaca,* and 3 species of *Papio*), colobines (2 species of *Presbytis*, 1 species of *Pygathrix*, and 3 species of *Trachypithecus*) and atelines (1 species of *Alouatta,* and 4 species of *Ateles*) (Fig. 3a and Appendix S3 online). Importantly, of the 121 primate species with very low (n = 74) and low (n = 47) behavioral flexibility, 109 (90%) are currently listed by the IUCN as threatened (Vulnerable, Endangered, or Critically Endangered) (Fig. 3a). This represents ~ 55% of all species assessed. In contrast, only ~ 8% of species with high or very high behavioral flexibility (14 and 3 species, respectively) were listed as threatened (See Appendix S3 online).

In Madagascar, of the 97 cyclone-exposed primate species, ~ 87% were assessed as having very low or low behavioral flexibility (Fig. 3b). Overall, 80 (95.2%) of these species were classified as threatened and only 4 as non-threatened. These species were predominantly, but not exclusively, woolly lemurs, sportive lemurs, and mouse lemurs (See Supplementary Appendix S3 online). In Asia, ~ 43% of the 68 cyclone-exposed primates (e.g., gibbons, langurs, macaques) were assessed as having very low and low behavioral flexibility (Fig. 3b), of which 24 (82.7%) were classified as threatened and 5 as non-threatened (See Supplementary Appendix S3 online). Furthermore, only 13 cyclone-exposed Asian primate species were scored as exhibiting high behavioral flexibility (gibbons, macaques, colobines), with 9 classified as threatened and 2 as non-threatened.

In contrast, in the Neotropics (Fig. 3b), only 6 of 21 cyclone-exposed primates were characterized by very low or low behavioral flexibility (*Callicebus personatus, Sapajus flavius*, *Alouatta arctoidea*, *Aotus griseimembra, Brachyteles hypoxanthus*, *Callithrix flaviceps*). Five cyclone-exposed Neotropical primates were characterized with high or very high behavioral flexibility (four subspecies of spider monkeys, *Ateles geoffroyi frontatus, A. g. geoffroyi, A. g. vellerosus, Ateles hybridus*, and one howler monkey *Alouatta seniculus juara*).

We identified 13 cyclone-exposed primate species in mainland Africa (Fig. 3b). Two were characterized with very low behavioral flexibility (*Galago gallarum* and *G. moholi*), 3 with moderate levels of behavioral flexibility (*Colobus angolensis*; *Galagoides granti* and *Otolemur garnettii*), and 8 with high behavioral flexibility (guenons, green monkeys, baboons). None of these 13 primate species are currently classified as threatened by the IUCN (See Appendix S3 online).

A closer examination of the 121 cyclone-exposed primates with low or very low flexibility shows that 87 are prosimians, including 2 African lorisids, 3 Asian lorisids, and 84 Malagasy lemurs. In the case of higher primates, 6 species of Neotropical monkeys, 18 species of Asian monkeys, and 8 species of Asian small apes (gibbons) were found to be cyclone exposed and have low behavioral flexibility. We note that one species of Asian monkey (*Trachypithecus phayrei*), 3 species of small apes (*Nomascus gabriellae, N. leucogenys, N. siki*), and 6 species of prosimians (*Avahi laniger*, *Cheirogaleus major*, *Eulemur rufifrons*, *Mirza coquereli*, *Phaner pallescens*, and *Carlito syrichta* (formerly *Tarsius syrichta*) have very high cyclone exposure. Each of these species are currently listed as threatened in the IUCN Red List except for *Carlito syrichta*, and therefore future increases in ECE are likely to expose them to a higher risk of extinction. Overall, we found that a greater proportion of primates exposed to cyclones in Madagascar (prosimians, 87%) and Asia (prosimians 5%, monkeys 26%, and small apes 12%) exhibited lower behavioral flexibility and increased exposure to cyclones, compared to primates in the Neotropics (monkeys 28%), and mainland Africa (prosimians, 14%).

## Discussion

Animals infrequently encounter extreme climatic events, however, those events are likely to have a disproportionate effect on species’ evolution, adaptability, and population persistence^25,33–35^. Recent changes in the frequency, intensity, and duration of ECEs^4^ mean that animal populations are facing greater environmental disturbances^36–42^. In this context, behavioral studies have examined species differences in response to environmental conditions caused by ECEs, including how changes to community structure and dynamics can have long-term negative consequences for predator–prey relationships, patterns of seed dispersal, and forest regeneration^3,17,43–45^. While much work remains to better understand the mechanisms driving differences in species’ behavioral flexibility to ECEs across several vertebrate lineages^17,46^, our framework provides a productive approach to link knowledge about species’ current biology and ecology, with flexibility in behaviors that can help to improve estimates of their current vulnerability to climate phenomena such as cyclones^20,38^. This knowledge can then better inform prioritization and conservation management strategies.

Nearly half of the primate species with the greatest cyclone exposure (mainly African and Asian cercopithecids, lemurs and atelines) exhibited a combination of attributes associated with high or very high behavioral flexibility. For instance, the Northern pig-tailed macaque (*Macaca leonina*), assessed with high behavioral flexibility, exploits multiple forest habitats across an elevational range of from 50 to 2000 m including tropical, bamboo, broadleaf, cloud forest, deciduous, dipterocarp, evergreen, gallery, lowland, montaine, primary, rainforest, riverine, secondary, and subtropical^47^. This species is reported to consume at least 10 different food types^47^ and exhibit fission–fusion dynamics during foraging^48^. This in turn, may allow the Northern pig-tailed macaque to buffer against habitat disruptions and short-term resource scarcity resulting from ECEs^49^.

Aye-ayes (*Daubentonia madagasarensis*), a Malagasy prosimian, lack fission–fusion dynamics but also rank among the most behaviorally flexible and cyclone-exposed primates. This species has the most widespread distribution of any extant lemur, and exploits nearly all forest strata across multiple ecosystems including heavily degraded forests^50^. These attributes have been associated with the aye-aye’s dietary flexibility, which includes the consumption of insect larvae, adult insects, seeds, nectar, and cankers (small areas of dead plant tissues)^51^. The aye-aye also is characterized by a highly diverse locomotor repertoire^51,52^. Unfortunately, the ongoing pace of human-induced habitat transformation across Madagascar (90% of Madagascar’s forests have been cut^53^), means that elements of behavioral flexibility selected for in response to ECEs, may no longer be sufficiently effective to ensure population persistence under conditions of extreme environmental degradation. Of the 105 species of Malagasy lemurs for which data are available, 98% are listed as threatened in the IUCN Red List^18^.

We identified that together with the aye-aye and the pig-tailed macaque, other primate taxa with high or very high behavioral flexibility (e.g., *Ateles geoffroyi frontatus*, *A. g. geoffroyi*, *Ateles g. vellerosus, Lemur catta*, *Hoolock hoolock*, *Pygathrix nigripes*, and *Trachypithecus germaini*) are currently categorized as threatened^54^. In the implementation of our framework, we are not using a species’ threatened status in the IUCN Red List to assign behavioral flexibility scores. Species attributes such as habitat type and elevational range, can be affected by a species conservation status and researchers should take this into account when using and expanding our framework. We focus on species’ current capacity for behaving flexibly as a result of present biological and ecological conditions. It is certainly true that an Endangered or Critically Endangered species, as assessed in the IUCN Red List, may no longer exploit certain habitats that it once exploited. However, it also is likely that the habitat in question has been altered or converted due to human activities and therefore the species is unlikely to encounter that habitat again after an ECE. This same situation for threatened primates is likely to exist in the case of other threatened taxa. That is why attributes associated with behavioral flexibility in diet and subgrouping are important in the framework as they are unrelated to a species IUCN Red List status.

We note some additional caveats that need to considered in adapting and applying this framework to other taxa. In absence of sufficient observational data, we used the expanding literature of species’ attributes associated with behavioral responses to climate change^9,17,32^ to identify attributes (habitat breadth, diet breadth, elevational range, and fission–fusion dynamics) relevant for primate species under exposure to cyclone-induced disturbance (Table 1). Each attribute was given equal weight in our scoring system. In reality, however, some attributes may have greater relevance than others in the face of the same type of ECE. Thus, documenting the types and magnitude of negative responses of species exposed (e.g., degree of population decline) to the same type of ECE and the behavioral characteristics found to be associated with these responses remain an important avenue for field investigations. In this regard, the framework can be modified to use additional attribute data and/or to give different weight to particular attributes based on specific knowledge of the behavior and biology of the taxa under study. This is critical to better inform the scoring and ranking of the target taxa. The reliability of assessments derived from this framework, as applied to other taxa and ECEs, will depend on the availability of empirical information on how the attributes examined enable individual species to cope with the newly created environmental conditions resulting from an ECE.

In the case of group-living primates, the ability to subgroup or exhibit flexibility in adjusting foraging group size to changes in food availability is a major advantage to survivorship under conditions in which group or population biomass rapidly exceeds food availability in the environment. This imbalance between group/population biomass and the resource availability in the face of ECEs is immediate and can be quite large^16^. Certainly solitary foragers require a smaller supply area and less food than group-living foragers. Quantitative data on species biomass and food availability prior to and after an ECE are critical for assessing the degree to which diet, habitat or species physiology best explain the ability of a population to recover after a particular ECE. We note however, that in other taxonomic groups, different behavioral characteristics may be more relevant to individuals or groups subjected to cyclones or other types of ECEs (e.g., huddling for thermoregulation or facultative migration in birds^55–57^).

We did not include torpor or hibernation in assessing behavioral flexibility of primates because these attributes are considered physiological adaptations to seasonality^58^. We acknowledge, however, that they could allow animals to cope with prolonged periods of food scarcity caused by cyclones or other ECEs provided appropriate hibernacula sites are still available. However, caution is needed in selecting these traits—no reports of torpor during cyclones are available for primates, yet, during torpor animal species may undergo a large absence of behavioral responses, sleep deprivation, increased predation due to immobility and/or inactivity, and increased risk of freezing^59^.

Species differences in certain life history characteristics (e.g., increased litter size, early age at first reproduction, shortened gestation length and interbirth interval, and reduced body mass), not directly related to behavioral flexibility, may also serve to offset or mute the ecological and survivorship challenges faced by recurring exposure to ECEs^60,61^. By reducing the time and resources necessary for individuals born after an ECE to reach sexually maturity, these life history characteristics may make it easier for a population to recover from the mortality of pre-reproductive young during an ECE.

For example, the black howler monkeys (*Alouatta pigra*) were assessed as exhibiting moderate behavioral flexibility to cyclones. Compared to spider monkeys (e.g., *Ateles g. vellerosus*) which were assessed as having very high behavioral flexibility and sympatric with black howler monkeys across part of their range, the black howlers have a shorter gestation length (average 173 days vs 230 days), shorter interbirth interval (average 19.5 months vs 32 months), and an earlier age at first reproduction (average 52 months vs 84 months)^62^. Given that howler monkeys lack the fission–fusion dynamics of spider monkeys, and thus may be less efficient in adapting to marked changes in the distribution and availability of scarce and scattered food patches following an ECE, their life history traits may help the population to recover more quickly following ECE induced mortality.

In addition, species characterized by small effective population size are more likely to become locally extinct due to demographic stochasticity, inbreeding, stochastic climatic conditions and prevailing anthropogenic pressures^38,60,63,64^. In 2013, cyclone Haiyan hit the island of Bohol, in the Philippines. This island contains a population of the Philippine tarsier (*Carlito syrichta*) assessed as having low behavioral flexibility. This cyclone caused a dramatic decrease in the species population density from 157 individuals/km^2^ in 2010 to 36 individuals/km^2^ in 2014, six months after the cyclone^30^. The cyclone, along with habitat loss and illegal hunting for the pet trade have significantly increased the local risk of tarsier population extirpation on Haiyan Island^65^.

The purpose of our study is not to understand how exposure to ECEs such as cyclones, over evolutionary time, may have selected for behavioral flexibility. Moreover, it would require a substantial phylogenetic analysis to determine whether the ancestor of a taxonomic group in question was also exposed to cyclones and also exhibited the same set of attributes. We focus on contemporary exposure to ECEs as this knowledge can better inform current species prioritization, conservation and management strategies.

The 1990–2021 time period we used in our analysis corresponds with high quality spatial data based on field surveys identifying the distribution of primate species^23^. Likewise, after the late 1980s, major improvements were made in the detection and estimation of cyclone activity including the use of Dvorak’s technique for cyclone intensity estimation based on infrared images^66^ and the development of coastal radars and advanced air-borne sensors^67,68^. Accordingly, by focusing on the time period for which cyclones’ and species’ occurrence are most reliable, we limit potential biases in the exposure analysis, capturing areas where primate populations currently occur and are exposed to cyclones.

We did not incorporate intensity and duration of ECEs in our framework because our aim is to select the species whose distribution has been exposed to cyclones in the recent past (1990–2021) rather than quantifying the severity of each cyclone to which species were exposed. Certainly, intensity and duration of an ECE and the local habitat conditions where it occurs can help to infer how harmful it may be for resident species populations. However, this is not as straightforward to undertake as it may initially seem. First, for cyclones, location-specific wind speed records are not available for each cyclone track or all of the segments in the trajectory of a given cyclone^66^; this makes it difficult to draw wind speed thresholds within the particular segment(s) of a cyclone track that overlay(s) the distribution of a given species. We expect that future improvements in modelling, forcasting and tracking ECEs will help mitigate this limitation. Second, the severity of a climatic event on a given species is likely to depend on differences in vegetation structure, habitat quality, and the horizontal zonation of affected populations relative to the source of disturbance^8^. Given that primates are facing an impending extinction crisis^18^, there is a growing interest in documenting the direct and indirect cyclone-related impacts to primate populations and their habitats^16,30,69–72^. Future research must focus on documenting how multiple groups/populations of the same species under these contrasting habitat conditions respond to the same ECEs^8^. With this information, researchers can test and validate the outcome of behavioral flexibility assessments for individual species against observed changes in population persistence and distribution in the face of more frequent and/or intense ECEs.

A hypothesis-driven quantitative analysis of attributes linked to behavioral flexibility in "response(s)" to disturbances caused by exposure to extreme climatic events (for example, one or more types of negative responses such as decline in body condition, fecundity, or increased mortality) is beyond the scope of our study. However, we encourage researchers studying animal responses to these events to collect such data^73,74^. In the short-term, changes in body condition, fecundity, or evidence of increased mortality represent the immediate consequences of ECEs. Monitoring the degree to which these conditions persist in the longer-term is essential for developing conservation strategies to protect vulnerable species and populations from extinction. As more attention is paid to how species are affected and respond to increases in the frequency and severity of ECEs, researchers will be able to better generate and test taxa-specific hypothesis concerning the set of attributes associated with species survival and population recovery.

In conclusion, we introduced a general framework to identify and distinguish between non-human primate species that have a limited capacity to anticipate, withstand and/or adjust their behavior in the face of ECEs and non-human primate species that have sufficient behavioral flexibility to more likely withstand the effects of ECEs. Although we applied this framework to one Order of mammals, we feel that it can be adapted to other animal species. We encourage wildlife managers and conservation practitioners to employ this framework in order to gain a greater understanding of the potential of different animal species to cope with extreme climatic events, in order to develop effective conservation and management policies to protect threatened species in the face of climate change.

### Supplementary Information

Supplementary Information.

## Data Availability

All data needed to revise the conclusions of this research are present in the paper and/or the supplementary materials online.
