# Supplementary material for: A conceptual framework for assessing behavioral flexibility of species in response to extreme climatic events
Source: Sci Rep. 2023 Oct 28;13:18478. doi: 10.1038/s41598-023-45756-2 (PMC10613232; doi:10.1038/s41598-023-45756-2)
Supplement: Supplementary file 1 — Supplementary Information. [file 41598_2023_45756_MOESM1_ESM.docx]

**Supplementary material available online**

Table S1 – Measure of confidence of attribute scores used to assess behavioral flexibility in response to cyclone disturbance of the 199 primate species. Overall, data completeness for the attributes of fission-fusion, habitat breadth, and diet breadth was high (98%, 95%, and 95%, respectively, of the total number of primate species assessed). Elevation range data was the least complete attribute (65% of total species assessed).

| **Score and confidence class** | **Description** |
| --- | --- |
| 2, Good | Attribute data available for a given primate species and derived from sources retrieved from specialized online databases and the peer-reviewed literature. |
| 1, Moderate | Attribute data not available for the assessed species but estimated by taking the average value of that attribute from congeneric species. These data were retrieved from specialized online databases and the peer-reviewed literature. |
| 0, Low | Data for that attribute were unavailable from either the assessed species or congeneric species. |

Appendix S1 – List of databases and research articles used to extract attribute data linked to behavioral flexibility of primate species (n = 199) in response to cyclone disturbance.

Online databases

IUCN. 2021. The IUCN Red List of Threatened Species. Version 2021-3. Accessed at <https://www.iucnredlist.org>. [Feb 2022].

Kate E. Jones, Jon Bielby, Marcel Cardillo, Susanne A. Fritz, Justin O'Dell, C. David L. Orme, Kamran Safi, Wes Sechrest, Elizabeth H. Boakes, Chris Carbone, Christina Connolly, Michael J. Cutts, Janine K. Foster, Richard Grenyer, Michael Habib, Christopher A. Plaster, Samantha A. Price, Elizabeth A. Rigby, Janna Rist, Amber Teacher, Olaf R. P. Bininda-Emonds, John L. Gittleman, Georgina M. Mace, and Andy Purvis. 2009. PanTHERIA: a species-level database of life history, ecology, and geography of extant and recently extinct mammals. Ecology 90:2648. Accessed at <http://esapubs.org/archive/ecol/E090/184/>. [March 2022].

Madagascar Lemurs portal. Accessed at <https://www.lemursportal.org>. [Feb 2022].

Myers, P., R. Espinosa, C. S. Parr, T. Jones, G. S. Hammond, and T. A. Dewey. 2022. The Animal Diversity Web (online). Accessed at <https://animaldiversity.org>. [Feb 2022].

New England Primate Conservancy. Accessed at <https://www.neprimateconservancy.org>. [March 2022].

Parr, C. S., N. Wilson, P. Leary, K. S. Schulz, K. Lans, L. Walley, J. A. Hammock, A. Goddard, J. Rice, M. Studer, J. T. G. Holmes, and R. J. Corrigan, Jr. 2014. The Encyclopedia of Life v2: Providing Global Access to Knowledge About Life on Earth. Biodiversity Data Journal 2: e1079, doi:10.3897/BDJ.2.e1079. Accessed at <https://eol.org/users/sign_in>. [March 2022]

Research articles and books

Gould, L. Lemur catta Ecology: What We Know and What We Need to Know. Lemurs, 255–274. doi:10.1007/978-0-387-34586-4_12

Mittermeier, R.A. and Wilson, D.E., 2013. Handbook of the mammals of the world: Vol. 3: Primates.

Rakotoniaina, J. H., Kappeler, P. M., Ravoniarimbinina, P., Pechouskova, E., Hämäläinen, A. M., Grass, J., ... & Kraus, C. (2016). Does habitat disturbance affect stress, body condition and parasitism in two sympatric lemurs?. Conservation Physiology, 4(1).

Rowe, N., & Myers, M. (Eds.). (2016). All the world's primates (Vol. 514). Charlestown: Pogonias Press. Accessed at <https://alltheworldsprimates.org/About.aspx>. [March 2022]

Thalmann, U., & Geissmann, T. (2006). Conservation Assessment of the Recently Described John Cleese's Woolly Lemur, Avahi cleesei (Lemuriformes, Indridae). Primate Conservation, 2006(21), 45-49.

Appendix S2 – Working examples. Assessing behavioral flexibility in response to cyclone disturbance for three selected primate species.

Example 1. The Mexican spider monkey (*Ateles geoffroyi vellerosus*) exhibits fission-fusion dynamics, and hence is assigned a score of “1” for having this attribute. In addition, the values for diet breadth (food categories = 5), habitat breadth (habitat types = 4), and elevational range (2,500 meters) of this subspecies reached the upper quartile of the distribution among the values of the 199 species under analysis. Therefore, each of these 3 attributes was assigned a score of “1”. Following the framework (Figure 1), scores were summed for each attribute and provide a maximum score of “4” which is associated with “very high” behavioral flexibility.

Example 2. The Hainan gibbon (*Nomascus hainanus*) does not exhibit fission-fusion dynamics and neither the values for habitat breadth (habitat types = 2), and elevational range (550 meters) reached the upper quartile of the distribution among the 199 species under analysis. However, its diet breadth values (6 food categories) reached the upper quartile. The overall sum of scores for each attribute is “1”, and hence the Hainan gibbon was assessed as having “low” behavioral flexibility.

Example 3. Verraux’s sifaka (*Propithecus verreauxi*) does not exhibit fission-fusion dynamics and consequently was assigned a score of “0” for that attribute. Its diet breadth and habitat breadth values (food categories = 6; habitat types = 3, respectively) reached the upper quartile of the distribution among the 199 species under analysis, and hence it was assigned a score of “1” for each. Following the framework, Verreaux’s sifaka was assigned an overall score of “2” and assessed as having “moderate” behavioral flexibility.

Appendix S3 – Dataset with the four species attributes for 199 primate species exposed to cyclones for the period January 1990 to December 2021 that were assessed using the behavioral flexibility framework.

| **Primate taxa** | **Region** | **Family** | IUCN Risk status | **Habitat_breadth** | **Elevation_range** | **Diet_**  **breadh** | **Fission_**  **fusion** | **Sum_Scores** | **BF_Class** |
| --- | --- | --- | --- | --- | --- | --- | --- | --- | --- |
| *Allocebus trichotis* | Madagascar | Cheirogaliidae | Threatened | 0 | 0 | 0 | 0 | 0 | Very Low |
| *Alouatta arctoidea* | Neotropics | Atelidae | Non-threatened | 1 | 0 | 0 | 0 | 1 | Low |
| *Alouatta guariba* | Neotropics | Atelidae | Non-threatened | 1 | 1 | 0 | 0 | 2 | Medium |
| *Alouatta macconnelli* | Neotropics | Atelidae | Non-threatened | 1 | 1 | 0 | 0 | 2 | Medium |
| *Alouatta palliata mexicana* | Neotropics | Atelidae | Non-threatened | 1 | 1 | 0 | 0 | 2 | Medium |
| *Alouatta palliata palliata* | Neotropics | Atelidae | Threatened | 1 | 1 | 0 | 0 | 2 | Medium |
| *Alouatta pigra* | Neotropics | Atelidae | Threatened | 1 | 1 | 0 | 0 | 2 | Medium |
| *Alouatta seniculus juara* | Neotropics | Atelidae | Non-threatened | 1 | 1 | 1 | 0 | 3 | High |
| *Aotus griseimembra* | Neotropics | Aotidae | Threatened | 1 | 0 | 0 | 0 | 1 | Low |
| *Ateles geoffroyi frontatus* | Neotropics | Atelidae | Threatened | 1 | 1 | 1 | 1 | 4 | Very High |
| *Ateles geoffroyi geoffroyi* | Neotropics | Atelidae | Threatened | 1 | 1 | 1 | 1 | 4 | Very High |
| *Ateles geoffroyi vellerosus* | Neotropics | Atelidae | Threatened | 1 | 1 | 1 | 1 | 4 | Very High |
| *Ateles hybridus* | Neotropics | Atelidae | Threatened | 1 | 0 | 1 | 1 | 3 | High |
| *Avahi betsileo* | Madagascar | Indriidae | Threatened | 0 | 0 | 0 | 0 | 0 | Very Low |
| *Avahi cleesei* | Madagascar | Indriidae | Threatened | 0 | 0 | 0 | 0 | 0 | Very Low |
| *Avahi laniger* | Madagascar | Indriidae | Threatened | 0 | 0 | 0 | 0 | 0 | Very Low |
| *Avahi meridionalis* | Madagascar | Indriidae | Threatened | 0 | 0 | 0 | 0 | 0 | Very Low |
| *Avahi mooreorum* | Madagascar | Indriidae | Threatened | 0 | 0 | 0 | 0 | 0 | Very Low |
| *Avahi occidentalis* | Madagascar | Indriidae | Threatened | 0 | 0 | 0 | 0 | 0 | Very Low |
| *Avahi peyrierasi* | Madagascar | Indriidae | Threatened | 0 | 0 | 0 | 0 | 0 | Very Low |
| *Avahi ramanantsoavanai* | Madagascar | Indriidae | Threatened | 0 | 0 | 0 | 0 | 0 | Very Low |
| *Avahi unicolor* | Madagascar | Indriidae | Threatened | 0 | 0 | 0 | 0 | 0 | Very Low |
| *Brachyteles hypoxanthus* | Neotropics | Atelidae | Threatened | 0 | 1 | 0 | 0 | 1 | Low |
| *Callicebus personatus* | Neotropics | Pitheciidae | Threatened | 0 | 0 | 0 | 0 | 0 | Very Low |
| *Callithrix flaviceps* | Neotropics | Callitrichidae | Threatened | 0 | 0 | 1 | 0 | 1 | Low |
| *Callithrix geoffroyi* | Neotropics | Callitrichidae | Non-threatened | 1 | 0 | 1 | 0 | 2 | Medium |
| *Carlito syrichta* | Asia | Tarsiidae | Non-threatened | 1 | 0 | 0 | 0 | 1 | Low |
| *Cebus albifrons* | Neotropics | Cebidae | Non-threatened | 1 | 0 | 1 | 0 | 2 | Medium |
| *Cercopithecus mitis* | Mainland_Africa | Cercopithecidae | Non-threatened | 1 | 1 | 1 | 0 | 3 | High |
| *Cheirogaleus crossleyi* | Madagascar | Cheirogaliidae | Threatened | 0 | 1 | 0 | 0 | 1 | Low |
| *Cheirogaleus major* | Madagascar | Cheirogaliidae | Threatened | 0 | 0 | 1 | 0 | 1 | Low |
| *Cheirogaleus medius* | Madagascar | Cheirogaliidae | Non-threatened | 0 | 0 | 1 | 0 | 1 | Low |
| *Cheirogaleus sibreei* | Madagascar | Cheirogaliidae | Threatened | 0 | 0 | 0 | 0 | 0 | Very Low |
| *Chlorocebus aethiops* | Mainland_Africa | Cercopithecidae | Non-threatened | 1 | 1 | 1 | 0 | 3 | High |
| *Chlorocebus pygerythrus* | Mainland_Africa | Cercopithecidae | Non-threatened | 1 | 1 | 1 | 0 | 3 | High |
| *Colobus angolensis* | Mainland_Africa | Cercopithecidae | Non-threatened | 0 | 1 | 1 | 0 | 2 | Medium |
| *Daubentonia madagascariensis* | Madagascar | Daubentoniidae | Threatened | 1 | 1 | 1 | 0 | 3 | High |
| *Eulemur albifrons* | Madagascar | Lemuridae | Threatened | 0 | 0 | 0 | 0 | 0 | Very Low |
| *Eulemur cinereiceps* | Madagascar | Lemuridae | Threatened | 0 | 0 | 1 | 1 | 2 | Medium |
| *Eulemur collaris* | Madagascar | Lemuridae | Threatened | 0 | 1 | 0 | 0 | 1 | Low |
| *Eulemur coronatus* | Madagascar | Lemuridae | Threatened | 1 | 0 | 1 | 1 | 3 | High |
| *Eulemur flavifrons* | Madagascar | Lemuridae | Threatened | 0 | 0 | 1 | 0 | 1 | Low |
| *Eulemur fulvus* | Madagascar | Lemuridae | Non-threatened | 1 | 1 | 1 | 0 | 3 | High |
| *Eulemur macaco* | Madagascar | Lemuridae | Threatened | 0 | 0 | 1 | 0 | 1 | Low |
| *Eulemur mongoz* | Madagascar | Lemuridae | Threatened | 0 | 0 | 0 | 0 | 0 | Very Low |
| *Eulemur rubriventer* | Madagascar | Lemuridae | Threatened | 0 | 1 | 1 | 0 | 2 | Medium |
| *Eulemur rufifrons* | Madagascar | Lemuridae | Non-threatened | 1 | 0 | 0 | 0 | 1 | Low |
| *Eulemur rufus* | Madagascar | Lemuridae | Threatened | 0 | 0 | 0 | 0 | 0 | Very low |
| *Eulemur sanfordi* | Madagascar | Lemuridae | Threatened | 1 | 0 | 0 | 0 | 1 | Low |
| *Galago gallarum* | Mainland_Africa | Galagidae | Non-threatened | 0 | 0 | 0 | 0 | 0 | Very Low |
| *Galago moholi* | Mainland_Africa | Galagidae | Non-threatened | 0 | 0 | 0 | 0 | 0 | Very Low |
| *Galago senegalensis* | Mainland_Africa | Galagidae | Non-threatened | 1 | 1 | 1 | 0 | 3 | High |
| *Galagoides granti* | Mainland_Africa | Galagidae | Non-threatened | 0 | 1 | 1 | 0 | 2 | Medium |
| *Hapalemur alaotrensis* | Madagascar | Lemuridae | Threatened | 0 | 0 | 0 | 0 | 0 | Very Low |
| *Hapalemur aureus* | Madagascar | Lemuridae | Threatened | 0 | 0 | 0 | 0 | 0 | Very Low |
| *Hapalemur griseus gilberti* | Madagascar | Lemuridae | Threatened | 0 | 1 | 1 | 0 | 2 | Medium |
| *Hapalemur griseus griseus* | Madagascar | Lemuridae | Threatened | 0 | 1 | 1 | 0 | 2 | Medium |
| *Hapalemur griseus ranamafanensis* | Madagascar | Lemuridae | Threatened | 0 | 1 | 1 | 0 | 2 | Medium |
| *Hapalemur meridionalis* | Madagascar | Lemuridae | Threatened | 0 | 0 | 0 | 0 | 0 | Very Low |
| *Hapalemur occidentalis* | Madagascar | Lemuridae | Threatened | 1 | 0 | 0 | 0 | 1 | Low |
| *Hoolock hoolock* | Asia | Hylobatidae | Threatened | 1 | 1 | 1 | 0 | 3 | High |
| *Hoolock leuconedys* | Asia | Hylobatidae | Threatened | 1 | 1 | 1 | 0 | 3 | High |
| *Hylobates agilis* | Asia | Hylobatidae | Threatened | 1 | 0 | 0 | 0 | 1 | Low |
| *Hylobates lar* | Asia | Hylobatidae | Threatened | 1 | 0 | 1 | 0 | 2 | Medium |
| *Hylobates muelleri* | Asia | Hylobatidae | Threatened | 0 | 0 | 0 | 0 | 0 | Very Low |
| *Hylobates pileatus* | Asia | Hylobatidae | Threatened | 1 | 0 | 1 | 0 | 2 | Medium |
| *Indri indri* | Madagascar | Indriidae | Threatened | 0 | 0 | 1 | 0 | 1 | Low |
| *Lemur catta* | Madagascar | Lemuridae | Threatened | 1 | 1 | 1 | 0 | 3 | High |
| *Lepilemur aeeclis* | Madagascar | Lepilemuridae | Threatened | 0 | 0 | 0 | 0 | 0 | Very Low |
| *Lepilemur ahmansonorum* | Madagascar | Lepilemuridae | Threatened | 0 | 0 | 0 | 0 | 0 | Very Low |
| *Lepilemur ankaranensis* | Madagascar | Lepilemuridae | Threatened | 0 | 0 | 0 | 0 | 0 | Very Low |
| *Lepilemur betsileo* | Madagascar | Lepilemuridae | Threatened | 0 | 0 | 0 | 0 | 0 | Very Low |
| *Lepilemur dorsalis* | Madagascar | Lepilemuridae | Threatened | 0 | 0 | 0 | 0 | 0 | Very Low |
| *Lepilemur edwardsi* | Madagascar | Lepilemuridae | Threatened | 0 | 0 | 1 | 0 | 1 | Low |
| *Lepilemur fleuretae* | Madagascar | Lepilemuridae | Threatened | 0 | 0 | 0 | 0 | 0 | Very Low |
| *Lepilemur grewcockorum* | Madagascar | Lepilemuridae | Threatened | 0 | 0 | 0 | 0 | 0 | Very Low |
| *Lepilemur hollandorum* | Madagascar | Lepilemuridae | Threatened | 0 | 0 | 0 | 0 | 0 | Very Low |
| *Lepilemur hubbardorum* | Madagascar | Lepilemuridae | Threatened | 0 | 0 | 0 | 0 | 0 | Very Low |
| *Lepilemur jamesorum* | Madagascar | Lepilemuridae | Threatened | 0 | 0 | 0 | 0 | 0 | Very Low |
| *Lepilemur leucopus* | Madagascar | Lepilemuridae | Threatened | 0 | 0 | 0 | 0 | 0 | Very Low |
| *Lepilemur microdon* | Madagascar | Lepilemuridae | Threatened | 0 | 0 | 0 | 0 | 0 | Very Low |
| *Lepilemur milanoii* | Madagascar | Lepilemuridae | Threatened | 0 | 0 | 0 | 0 | 0 | Very Low |
| *Lepilemur mittermeieri* | Madagascar | Lepilemuridae | Threatened | 0 | 0 | 0 | 0 | 0 | Very Low |
| *Lepilemur mustelinus* | Madagascar | Lepilemuridae | Non-threatened | 0 | 0 | 0 | 0 | 0 | Very Low |
| *Lepilemur otto* | Madagascar | Lepilemuridae | Threatened | 0 | 0 | 0 | 0 | 0 | Very Low |
| *Lepilemur petteri* | Madagascar | Lepilemuridae | Threatened | 0 | 0 | 0 | 0 | 0 | Very Low |
| *Lepilemur randrianasoloi* | Madagascar | Lepilemuridae | Threatened | 0 | 0 | 0 | 0 | 0 | Very Low |
| *Lepilemur ruficaudatus* | Madagascar | Lepilemuridae | Threatened | 0 | 0 | 0 | 0 | 0 | Very Low |
| *Lepilemur sahamalazensis* | Madagascar | Lepilemuridae | Threatened | 1 | 0 | 0 | 0 | 1 | Low |
| *Lepilemur scottorum* | Madagascar | Lepilemuridae | Threatened | 0 | 0 | 0 | 0 | 0 | Very Low |
| *Lepilemur seali* | Madagascar | Lepilemuridae | Threatened | 1 | 0 | 0 | 0 | 1 | Low |
| *Lepilemur septentrionalis* | Madagascar | Lepilemuridae | Threatened | 0 | 0 | 0 | 0 | 0 | Very Low |
| *Lepilemur tymerlachsoni* | Madagascar | Lepilemuridae | Threatened | 0 | 0 | 0 | 0 | 0 | Very Low |
| *Lepilemur wrightae* | Madagascar | Lepilemuridae | Threatened | 0 | 0 | 0 | 0 | 0 | Very Low |
| *Loris lydekkerianus* | Asia | Lorisidae | Non-threatened | 1 | 1 | 1 | 0 | 3 | High |
| *Loris tardigradus* | Asia | Lorisidae | Threatened | 1 | 1 | 0 | 0 | 2 | Medium |
| *Macaca arctoides* | Asia | Cercopithecidae | Threatened | 0 | 1 | 1 | 0 | 2 | Medium |
| *Macaca assamensis* | Asia | Cercopithecidae | Non-threatened | 0 | 1 | 1 | 0 | 2 | Medium |
| *Macaca cyclopis* | Asia | Cercopithecidae | Non-threatened | 1 | 1 | 1 | 0 | 3 | High |
| *Macaca fascicularis philippensis* | Asia | Cercopithecidae | Non-threatened | 1 | 1 | 0 | 0 | 2 | Medium |
| *Macaca fuscata* | Asia | Cercopithecidae | Non-threatened | 0 | 0 | 1 | 0 | 1 | Low |
| *Macaca leonina* | Asia | Cercopithecidae | Threatened | 1 | 1 | 1 | 0 | 3 | High |
| *Macaca mulatta* | Asia | Cercopithecidae | Non-threatened | 1 | 1 | 1 | 0 | 3 | High |
| *Macaca nemestrina* | Asia | Cercopithecidae | Threatened | 1 | 1 | 1 | 0 | 3 | High |
| *Macaca radiata* | Asia | Cercopithecidae | Non-threatened | 1 | 0 | 1 | 0 | 2 | Medium |
| *Macaca silenus* | Asia | Cercopithecidae | Threatened | 1 | 0 | 1 | 0 | 2 | Medium |
| *Macaca sinica* | Asia | Cercopithecidae | Threatened | 1 | 1 | 1 | 0 | 3 | High |
| *Macaca thibetana* | Asia | Cercopithecidae | Non-threatened | 1 | 0 | 1 | 0 | 2 | Medium |
| *Microcebus berthae* | Madagascar | Cheirogaliidae | Threatened | 0 | 0 | 0 | 0 | 0 | Very Low |
| *Microcebus bongolavensis* | Madagascar | Cheirogaliidae | Threatened | 0 | 0 | 1 | 0 | 1 | Low |
| *Microcebus danfossi* | Madagascar | Cheirogaliidae | Threatened | 0 | 0 | 0 | 0 | 0 | Very Low |
| *Microcebus griseorufus* | Madagascar | Cheirogaliidae | Non-threatened | 0 | 0 | 0 | 0 | 0 | Very Low |
| *Microcebus jollyae* | Madagascar | Cheirogaliidae | Threatened | 0 | 0 | 0 | 0 | 0 | Very Low |
| *Microcebus lehilahytsara* | Madagascar | Cheirogaliidae | Threatened | 0 | 0 | 0 | 0 | 0 | Very Low |
| *Microcebus macarthurii* | Madagascar | Cheirogaliidae | Threatened | 0 | 0 | 0 | 0 | 0 | Very Low |
| *Microcebus mamiratra* | Madagascar | Cheirogaliidae | Threatened | 0 | 0 | 0 | 0 | 0 | Very Low |
| *Microcebus margotmarshae* | Madagascar | Cheirogaliidae | Threatened | 0 | 0 | 0 | 0 | 0 | Very Low |
| *Microcebus mittermeieri* | Madagascar | Cheirogaliidae | Threatened | 0 | 0 | 0 | 0 | 0 | Very Low |
| *Microcebus murinus* | Madagascar | Cheirogaliidae | Non-threatened | 1 | 0 | 1 | 0 | 2 | Medium |
| *Microcebus myoxinus* | Madagascar | Cheirogaliidae | Threatened | 0 | 0 | 0 | 0 | 0 | Very Low |
| *Microcebus ravelobensis* | Madagascar | Cheirogaliidae | Threatened | 0 | 0 | 0 | 0 | 0 | Very Low |
| *Microcebus rufus* | Madagascar | Cheirogaliidae | Threatened | 1 | 0 | 1 | 0 | 2 | Medium |
| *Microcebus sambiranensis* | Madagascar | Cheirogaliidae | Threatened | 1 | 0 | 0 | 0 | 1 | Low |
| *Microcebus simmonsi* | Madagascar | Cheirogaliidae | Threatened | 0 | 0 | 0 | 0 | 0 | Very Low |
| *Microcebus tavaratra* | Madagascar | Cheirogaliidae | Threatened | 1 | 0 | 0 | 0 | 1 | Low |
| *Mirza coquereli* | Madagascar | Cheirogaliidae | Threatened | 0 | 0 | 0 | 0 | 0 | Very Low |
| *Mirza zaza* | Madagascar | Cheirogaliidae | Threatened | 0 | 0 | 1 | 0 | 1 | Low |
| *Nasalis larvatus* | Asia | Cercopithecidae | Threatened | 1 | 0 | 1 | 0 | 2 | Medium |
| *Nomascus concolor* | Asia | Hylobatidae | Threatened | 0 | 1 | 1 | 0 | 2 | Medium |
| *Nomascus gabriellae* | Asia | Hylobatidae | Threatened | 0 | 0 | 0 | 0 | 0 | Very Low |
| *Nomascus hainanus* | Asia | Hylobatidae | Threatened | 0 | 0 | 1 | 0 | 1 | Low |
| *Nomascus leucogenys* | Asia | Hylobatidae | Threatened | 0 | 0 | 0 | 0 | 0 | Very Low |
| *Nomascus nasutus* | Asia | Hylobatidae | Threatened | 0 | 0 | 1 | 0 | 1 | Low |
| *Nomascus siki* | Asia | Hylobatidae | Threatened | 0 | 0 | 0 | 0 | 0 | Very Low |
| *Nycticebus bengalensis* | Asia | Lorisidae | Threatened | 0 | 1 | 1 | 0 | 2 | Medium |
| *Nycticebus coucang* | Asia | Lorisidae | Threatened | 1 | 0 | 1 | 0 | 2 | Medium |
| *Nycticebus menagensis* | Asia | Lorisidae | Threatened | 0 | 0 | 0 | 0 | 0 | Very Low |
| *Nycticebus pygmaeus* | Asia | Lorisidae | Threatened | 1 | 0 | 1 | 0 | 2 | Medium |
| *Otolemur crassicaudatus* | Mainland_Africa | Galagidae | Non-threatened | 1 | 1 | 1 | 0 | 3 | High |
| *Otolemur garnettii* | Mainland_Africa | Galagidae | Non-threatened | 1 | 1 | 0 | 0 | 2 | Medium |
| *Papio cynocephalus* | Mainland_Africa | Cercopithecidae | Non-threatened | 1 | 1 | 1 | 0 | 3 | High |
| *Papio hamadryas* | Mainland_Africa | Cercopithecidae | Non-threatened | 1 | 1 | 1 | 0 | 3 | High |
| *Papio ursinus* | Mainland_Africa | Cercopithecidae | Non-threatened | 1 | 1 | 1 | 0 | 3 | High |
| *Phaner electromontis* | Madagascar | Cheirogaliidae | Threatened | 1 | 0 | 0 | 0 | 1 | Low |
| *Phaner furcifer* | Madagascar | Cheirogaliidae | Threatened | 0 | 0 | 0 | 0 | 0 | Very Low |
| *Phaner pallescens* | Madagascar | Cheirogaliidae | Threatened | 0 | 0 | 0 | 0 | 0 | Very Low |
| *Phaner parienti* | Madagascar | Cheirogaliidae | Threatened | 0 | 0 | 0 | 0 | 0 | Very Low |
| *Pongo abelii* | Asia | Hominidae | Threatened | 1 | 0 | 1 | 0 | 2 | Medium |
| *Presbytis femoralis* | Asia | Cercopithecidae | Non-threatened | 1 | 0 | 1 | 0 | 2 | Medium |
| *Presbytis hosei* | Asia | Cercopithecidae | Threatened | 1 | 0 | 1 | 0 | 2 | Medium |
| *Presbytis melalophos* | Asia | Cercopithecidae | Threatened | 1 | 0 | 0 | 0 | 1 | Low |
| *Presbytis natunae* | Asia | Cercopithecidae | Threatened | 0 | 0 | 1 | 0 | 1 | Low |
| *Presbytis rubicunda* | Asia | Cercopithecidae | Non-threatened | 1 | 1 | 1 | 0 | 3 | High |
| *Presbytis siamensis* | Asia | Cercopithecidae | Non-threatened | 1 | 0 | 0 | 0 | 1 | Low |
| *Presbytis thomasi* | Asia | Cercopithecidae | Threatened | 1 | 1 | 1 | 0 | 3 | High |
| *Prolemur simus* | Madagascar | Lemuridae | Threatened | 1 | 0 | 0 | 0 | 1 | Low |
| *Propithecus candidus* | Madagascar | Indriidae | Threatened | 0 | 0 | 1 | 0 | 1 | Low |
| *Propithecus coquereli* | Madagascar | Indriidae | Threatened | 0 | 0 | 0 | 0 | 0 | Very Low |
| *Propithecus coronatus* | Madagascar | Indriidae | Threatened | 0 | 0 | 0 | 0 | 0 | Very Low |
| *Propithecus deckenii* | Madagascar | Indriidae | Threatened | 0 | 0 | 0 | 0 | 0 | Very Low |
| *Propithecus diadema* | Madagascar | Indriidae | Threatened | 0 | 0 | 0 | 0 | 0 | Very Low |
| *Propithecus edwardsi* | Madagascar | Indriidae | Threatened | 0 | 0 | 1 | 0 | 1 | Low |
| *Propithecus perrieri* | Madagascar | Indriidae | Threatened | 0 | 0 | 0 | 0 | 0 | Very Low |
| *Propithecus tattersalli* | Madagascar | Indriidae | Threatened | 1 | 0 | 0 | 0 | 1 | Low |
| *Propithecus verreauxi* | Madagascar | Indriidae | Threatened | 1 | 0 | 1 | 0 | 2 | Medium |
| *Pygathrix cinerea* | Asia | Cercopithecidae | Threatened | 1 | 0 | 0 | 0 | 1 | Low |
| *Pygathrix nemaeus* | Asia | Cercopithecidae | Threatened | 0 | 1 | 0 | 1 | 2 | Medium |
| *Pygathrix nigripes* | Asia | Cercopithecidae | Threatened | 1 | 1 | 0 | 1 | 3 | High |
| *Rhinopithecus avunculus* | Asia | Cercopithecidae | Threatened | 0 | 0 | 0 | 0 | 0 | Very Low |
| *Rhinopithecus roxellana* | Asia | Cercopithecidae | Threatened | 0 | 0 | 1 | 0 | 1 | Low |
| *Saguinus oedipus* | Neotropics | Callitrichidae | Threatened | 1 | 0 | 1 | 0 | 2 | Medium |
| *Sapajus flavius* | Neotropics | Cebidae | Threatened | 0 | 0 | 0 | 0 | 0 | Very Low |
| *Sapajus nigritus* | Neotropics | Cebidae | Non-threatened | 1 | 0 | 1 | 0 | 2 | Medium |
| *Sapajus robustus* | Neotropics | Cebidae | Threatened | 1 | 0 | 1 | 0 | 2 | Medium |
| *Semnopithecus dussumieri* | Asia | Cercopithecidae | Non-threatened | 1 | 0 | 0 | 0 | 1 | Low |
| *Semnopithecus entellus* | Asia | Cercopithecidae | Non-threatened | 1 | 0 | 1 | 0 | 2 | Medium |
| *Semnopithecus hector* | Asia | Cercopithecidae | Non-threatened | 1 | 0 | 1 | 0 | 2 | Medium |
| *Semnopithecus hypoleucos* | Asia | Cercopithecidae | Threatened | 1 | 0 | 0 | 0 | 1 | Low |
| *Semnopithecus priam* | Asia | Cercopithecidae | Non-threatened | 1 | 0 | 1 | 0 | 2 | Medium |
| *Semnopithecus schistaceus* | Asia | Cercopithecidae | Non-threatened | 1 | 0 | 1 | 0 | 2 | Medium |
| *Symphalangus syndactylus* | Asia | Hylobatidae | Threatened | 0 | 1 | 0 | 0 | 1 | Low |
| *Tarsius bancanus* | Asia | Tarsiidae | Threatened | 1 | 0 | 0 | 0 | 1 | Low |
| *Trachypithecus auratus* | Asia | Cercopithecidae | Threatened | 1 | 1 | 1 | 0 | 3 | High |
| *Trachypithecus barbei* | Asia | Cercopithecidae | Threatened | 0 | 0 | 0 | 0 | 0 | Very Low |
| *Trachypithecus cristatus* | Asia | Cercopithecidae | Non-threatened | 1 | 0 | 0 | 0 | 1 | Low |
| *Trachypithecus delacouri* | Asia | Cercopithecidae | Threatened | 1 | 0 | 0 | 0 | 1 | Low |
| *Trachypithecus francoisi* | Asia | Cercopithecidae | Threatened | 1 | 0 | 1 | 0 | 2 | Medium |
| *Trachypithecus germaini* | Asia | Cercopithecidae | Threatened | 1 | 0 | 1 | 1 | 3 | High |
| *Trachypithecus hatinhensis* | Asia | Cercopithecidae | Threatened | 1 | 0 | 0 | 0 | 1 | Low |
| *Trachypithecus johnii* | Asia | Cercopithecidae | Threatened | 0 | 0 | 1 | 0 | 1 | Low |
| *Trachypithecus laotum* | Asia | Cercopithecidae | Threatened | 1 | 0 | 0 | 0 | 1 | Low |
| *Trachypithecus obscurus* | Asia | Cercopithecidae | Non-threatened | 1 | 0 | 1 | 0 | 2 | Medium |
| *Trachypithecus phayrei* | Asia | Cercopithecidae | Threatened | 1 | 0 | 0 | 0 | 1 | Low |
| *Trachypithecus pileatus* | Asia | Cercopithecidae | Threatened | 0 | 1 | 1 | 0 | 2 | Medium |
| *Trachypithecus poliocephalus leucocephalus* | Asia | Cercopithecidae | Threatened | 0 | 0 | 0 | 0 | 0 | Very Low |
| *Trachypithecus poliocephalus poliocephalus* | Asia | Cercopithecidae | Threatened | 0 | 0 | 0 | 0 | 0 | Very Low |
| *Trachypithecus vetulus* | Asia | Cercopithecidae | Threatened | 1 | 1 | 1 | 0 | 3 | High |
| *Varecia rubra* | Madagascar | Lemuridae | Threatened | 0 | 0 | 0 | 1 | 1 | Low |
| *Varecia variegata* | Asia | Lemuridae | Threatened | 0 | 0 | 1 | 1 | 2 | Medium |
